# Trends in peripheral artery disease and critical limb ischemia hospitalizations among cocaine and methamphetamine users in the United States: a nationwide study

**DOI:** 10.3389/fcvm.2024.1412867

**Published:** 2024-07-03

**Authors:** Shafaqat Ali, Zaki Al-Yafeai, Md. Ismail Hossain, Md. Shenuarin Bhuiyan, Sanchit Duhan, Richa Aishwarya, Nicholas E. Goeders, Md. Mostafizur Rahman Bhuiyan, Steven A. Conrad, John A. Vanchiere, A. Wayne Orr, Christopher G. Kevil, Mohammad Alfrad Nobel Bhuiyan

**Affiliations:** ^1^ Department of Medicine, Louisiana State University Health Sciences Center at Shreveport, Shreveport, LA, United States; ^2^ Department of Pathology and Translational Pathobiology, Louisiana State University Health Sciences Center at Shreveport, Shreveport, LA, United States; ^3^ Department of Molecular and Cellular Physiology, Louisiana State University Health Sciences Center at Shreveport, Shreveport, LA, United States; ^4^ Department of Medicine, Sinai Hospital of Baltimore, Baltimore, MD, United States; ^5^ Department of Pharmacology, Toxicology & Neuroscience, Louisiana State University Health Sciences Center at Shreveport, Shreveport, LA, United States; ^6^ Department of Psychiatry and Behavioral Medicine, Louisiana State University Health Sciences Center at Shreveport, Shreveport, LA, United States; ^7^ Louisiana Addiction Research Center, Louisiana State University Health Sciences Center at Shreveport, Shreveport, LA, United States; ^8^ Department of Pediatric Cardiology, Bangabandhu Sheikh Mujib Medical University, Dhaka, Bangladesh; ^9^ Department of Pediatrics, Louisiana State University Health Sciences Center at Shreveport, Shreveport, LA, United States

**Keywords:** critical limb ischemia, peripheral arterial disease, cocaine, methamphetamine, trends

## Abstract

**Background:**

Peripheral artery disease (PAD) is on the rise worldwide, ranking as the third leading cause of atherosclerosis-related morbidity; much less is known about its trends in hospitalizations among methamphetamine and cocaine users.

**Objectives:**

We aim to evaluate the overall trend in the prevalence of hospital admission for PAD with or without the use of stimulant abuse (methamphetamine and cocaine) across the United States. Additionally, we evaluated the PAD-related hospitalizations trend stratified by age, race, sex, and geographic location.

**Methods:**

We used the National Inpatient Sample (NIS) database from 2008 to 2020. The Cochran Armitage trend test was used to compare the trend between groups. Multivariate logistic regression was used to examine adjusted odds for PAD and CLI hospitalizations among methamphetamine and cocaine users.

**Results:**

Between 2008 and 2020, PAD-related hospitalizations showed an increasing trend in Hispanics, African Americans, and western states, while a decreasing trend in southern and Midwestern states (*p*-trend <0.05). Among methamphetamine users, an overall increasing trend was observed in men, women, western, southern, and midwestern states (*p*-trend <0.05). However, among cocaine users, PAD-related hospitalization increased significantly for White, African American, age group >64 years, southern and western states (*p*-trend <0.05). Overall, CLI-related hospitalizations showed an encouraging decreasing trend in men and women, age group >64 years, and CLI-related amputations declined for women, White patient population, age group >40, and all regions (*p*-trend <0.05). However, among methamphetamine users, a significantly increasing trend in CLI-related hospitalization was seen in men, women, White & Hispanic population, age group 26–45, western, southern, and midwestern regions.

**Conclusions:**

There was an increasing trend in PAD-related hospitalizations among methamphetamine and cocaine users for both males and females. Although an overall decreasing trend in CLI-related hospitalization was observed for both genders, an up-trend in CLI was seen among methamphetamine users. The upward trends were more prominent for White, Hispanic & African Americans, and southern and western states, highlighting racial and geographic variations over the study period.

## Introduction

Peripheral arterial disease (PAD) is an obstructive atherosclerotic and microvascular disease mainly affecting vessels in the lower extremities (1–4). PAD is well recognized as an important cause of cardiovascular morbidity and mortality, affecting more than 230 million adults worldwide and 8–12 million adults in the US (4–6). Critical limb ischemia (CLI), a severe stage of PAD, is associated with a high amputation rate and manifested by resting pain and non-healing wounds (7). The global and national prevalence continues to rise, mainly affecting people above 65 (5). PAD is known to be a significant cause of non-traumatic amputation in the US, significantly impacting patients and healthcare systems (8). In addition, PAD is the third most common clinical manifestation of atherosclerosis after coronary artery disease (CAD) and stroke (1, 9). However, PAD has traditionally been understudied compared to other atherosclerotic diseases, leading to significant lags in overall awareness regarding PAD-related hospitalizations and essential geographical, ethnic, and sex-based disparities in the US and worldwide (1).

Substance use is among the modifiable risk factors associated with atherosclerotic cardiovascular disease (ASCVD) (10–13). Despite tremendous efforts to legalize some drugs and stem the availability of illegal drugs, substance use is rising. Methamphetamine and cocaine use are strongly associated with ASCVD development and progression (10, 14, 15). Yet the underlying mechanisms remain elusive (16). Surprisingly, the biological and clinical effects of methamphetamine and cocaine on PAD have received considerably little attention (10, 17).

In contrast to coronary and cerebrovascular diseases, the geographical, ethnic, and socioeconomic variations among PAD patients are less well-defined. While prior studies showed a marked prevalence of PAD among the non-Hispanic Black population compared to the non-Hispanic White and non-Hispanic population, recent research has contradicted this, demonstrating a difference of less than 10% (1, 3, 18, 19). However, the risk of non-traumatic amputation is 20% higher among non-Hispanic Black than non-Hispanic White patients (19). Prior studies have reported PAD to be a male-predominant disease, with one study reporting PAD incidence to be 50% lower in women than men among VA patients (19). The geographical and temporal association data for PAD-related hospitalizations in the US is minimal. While the overall rate of non-traumatic lower extremity amputations declined between 2000 and 2008, compared to the South Atlantic region, the rates of amputation were generally higher in East South Central and West South Central and lower in the Middle Atlantic region (20). However, temporal and geographical trends for PAD in the US are poorly characterized.

To address this knowledge deficit, we investigate the temporal, geographical, and sociodemographic trends in PAD-related hospitalizations in the general population as well as among cocaine and methamphetamine users in the US (2008–2020).

## Methods

### Study design and population

The Healthcare Cost and Utilization Project (HCUP) created the extensive deidentified National Inpatient Database (NIS) database. With more than 7 million hospital admissions annually, the NIS is one of the nation's most extensive and most used healthcare databases. Since more states have recently joined the HCUP, the NIS now covers −97% of all Americans. The information is gathered from hospitals throughout all 50 states. The HCUP website provides access to the NIS data, freely available to the public. The standard International Classification of Disease Clinical Modifications codes were used to identify all adults (>18 years of age) hospitalized for PAD, CLI, methamphetamine, or cocaine use. A complete list of the ICD 9 and 10 codes for PAD, CLI, amputation, methamphetamine, and cocaine use and the states in each region are presented in Supplementary Tables S1–S3, respectively. Hospitalizations related to PAD or CLI were defined as instances where patients were admitted to the hospital with PAD or CLI listed as primary or secondary diagnoses during their index hospitalization. Traumatic amputations and users of any other substances were omitted from the data we collected; only non-traumatic amputations, methamphetamine, and cocaine users were identified and retained for analysis. The NIS database was deidentified; hence, our study was exempt from requiring an institutional review board (IRB) approval. Helsinki guideline was followed for reporting the results.

### Study outcomes

The primary outcome of the study was demographic, racial, and regional trends of PAD-related hospitalizations in the general population of cocaine and methamphetamine users. The secondary outcomes included demographic, racial, and regional trends of CLI-related hospitalizations and amputations among the general population, cocaine and methamphetamine users in the US from 2008 to 2020.

### Statistical analysis

We examined the trends of hospital admissions due to PAD in general and PAD among methamphetamine and cocaine users separately. Similar trends of hospital admissions for CLI and amputations in the general population, methamphetamine and cocaine users among US adults categorized using sociodemographic characteristics based on 2008–2020 NIS data. Trend analysis was conducted to test the rates of hospital admissions, and the Cochran Armitage trend test was used to compare the trend between groups. We used hospital and patient-level weights to calculate the national estimates. Descriptive statistics were used to summarize continuous and categorical variables. The frequency of missing values was summarized, and *Little's MCAR (missing completely at random)* was used to screen for missing data patterns. A non-significant *p*-value (*P* > 0.05) represented randomly missing, while a significant *P*-value (*P* < 0.05) indicated missing not at random (MNAR). Variables with missing data were identified, and overall missing data was less than 5%; we marked it missing and excluded it from the analysis. Multivariate logistic regression was used to examine adjusted odds for PAD and CLI hospitalizations among methamphetamine and cocaine users between 2008 and 2020. Covariates were selected based on clinical correlation with PAD and CLI. We utilized univariate screening for building the regression model; *p*-value <0.2 was used as cut off for the covariates to be included in the final regression analysis ref. The multicollinearity among independent variables was assessed by measuring the variance inflation factor (VIF) and tolerance (1/VIF). VIF ≥5 and tolerance value ≤0.2 was taken as a marker of significant correlation among independent variables ref. We included more than 18 covariates for multivariate analysis, listed in Supplementary Table S4. All analyses used the open-source software R (version 4.3.1) to account for NIS complex sample design and sample weights. *P* < 0.05 (2-tailed) was considered statistically significant for each analysis.

## Results

**Figure 1:**
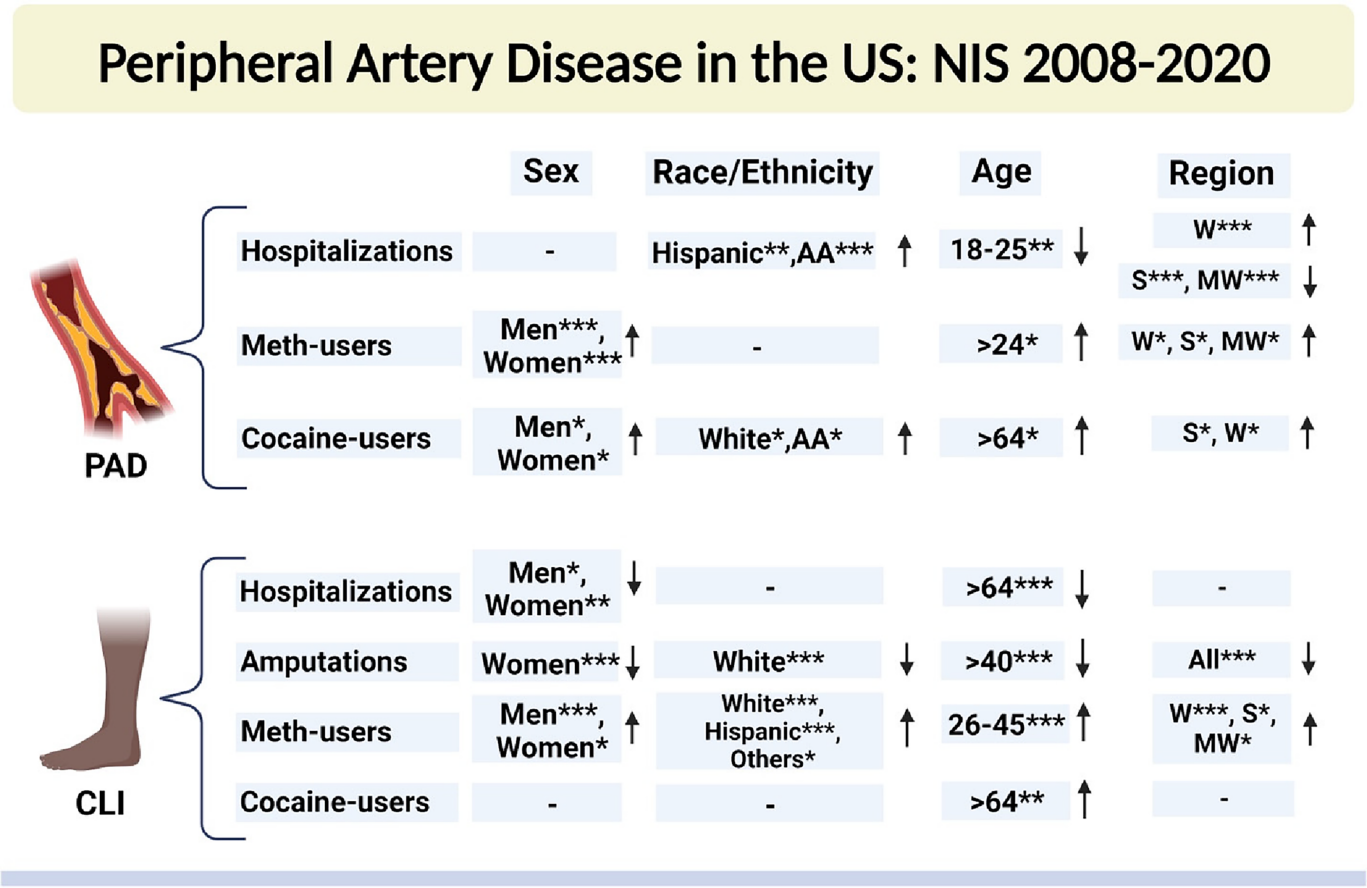
Sex-based, racial, ethical, Age-based, and region-based prevalence trends of PAD- and CLI-related hospitalizations and amputations between 2008 and 2020. NIS, national inpatient sample database; PAD, peripheral arterial disease; CLI, critical limb ischemia; AA, African American; W, west; S, south; MW, midwest. Che-square test was used to compare the categorical and t-test was used to compare the numerical values. *P < 0.05, **P < 0.01, and ***P < 0.001. ↑, increasing trend; ↓, decreasing trend; “−”, no significant change. Created with Biorender.com.

We identified 6,436,612 patients with peripheral arterial disease (PAD), 1,591,291 patients with critical limb ischemia (CLI), 8,011 patients with PAD among methamphetamine users, and 13,753 patients with PAD among cocaine users admitted to US hospitals between the years 2008 and 2020 using the National Inpatient Sample (NIS) database. Overall, Sex-based, Racial, Ethical, Age-based, and Region-based prevalence trends of PAD and CLI-related hospitalizations and amputations between 2008 and 2020 are shown in Figure 1.

### Baseline comorbidities and demographic characteristics of PAD patients

Our study population was predominantly male, particularly in groups with concomitant drug use: 53% vs. 46.9% among PAD patients, 59.32% vs. 40.68% among CLI patients, 63% vs. 37% in PAD patients with methamphetamine use, and 66.22% vs. 33.78% in PAD patients with cocaine use. Among these, 72.9% and 65.36% were over the age of 65 years in the PAD and CLI groups, respectively, while patients with PAD among methamphetamine and cocaine users were predominantly 41–64 years old (64.09% and 77.1%, respectively). The White patient population was predominant among PAD (70.9%), CLI (63.94%), and PAD in methamphetamine users (73.04%), while the Black patient population was predominant among PAD in cocaine users (63.87%). Baseline comorbidities and demographics are shown in Table 1.

**Table 1:** Demographic characteristics of PAD, CLI, who were also diagnosed as methamphetamine and cocaine users aged 18 or higher from 2008 to 2020 in the US.

Characteristics	Group	PAD, *n* (%) (95% CI)	CLI *n* (%) (95% CI)	PAD among Meth users *n* (%) (95% CI)	PAD among Cocaine users *n* (%) (95% CI)
NIS data 2008–2020	*N*	6,436,612	1,591,291	8,011	13,753
Sex	Men	3,413,485 (53.04) (53–53.08)	943,862 (59.32) (59.24–59.4)	5,043 (62.99) (61.93–64.05)	9,107 (66.22) (65.43–67.01)
	Women	3,022,487 (46.96) (46.92–47)	647,295 (40.68) (40.6–40.76)	2,963 (37.01) (35.95–38.07)	4,646 (33.78) (32.99–34.57)
Age (years)	Mean, SD	72, 12.02	69.61, 12.37	59.76, 11.85	56.25, 9.13
	18–25	5,101 (0.08) (0.08–0.08)	623 (0.04) (0.04–0.04)	26 (0.32) (0.2–0.44)	45 (0.33) (0.23–0.43)
	26–40	57,175 (0.89) (0.88–0.9)	17,708 (1.11) (1.1–1.13)	383 (4.78) (4.32–5.25)	620 (4.51) (4.16–4.85)
	41–64	1,681,929 (26.13) (26.1–26.16)	532,907 (33.49) (33.42–33.56)	5,134 (64.09) (63.04–65.14)	10,615 (77.19) (76.48–77.89)
	≥65	4,692,406 (72.9) (72.87–72.94)	1,040,053 (65.36) (65.29–65.43)	2,468 (30.81) (29.8–31.82)	2,473 (17.98) (17.34–18.62)
Race/Ethnicity^a^	White	4,291,992 (70.93) (70.89–70.97)	952,244 (63.94) (63.86–64.01)	5,607 (73.04) (72.04–74.03)	3,322 (25.31) (24.57–26.05)
	Black	847,534 (14.01) (13.98–14.03)	314,319 (21.1) (21.04–21.17)	633 (8.25) (7.63–8.86)	8,382 (63.87) (63.04–64.69)
	Hispanic	558,265 (9.23) (9.2–9.25)	150,330 (10.09) (10.05–10.14)	873 (11.38) (10.67–12.09)	964 (7.35) (6.9–7.79)
	Asian or Pacific Islander	187,212 (3.09) (3.08–3.11)	22,414 (1.5) (1.49–1.52)	285 (3.71) (3.28–4.13)	71 (0.54) (0.42–0.67)
	Native American	35,024 (0.58) (0.57–0.58)	11,113 (0.75) (0.73–0.76)	105 (1.37) (1.11–1.63)	79 (0.6) (0.47–0.74)
	Other	131,060 (2.17) (2.15–2.18)	38,964 (2.62) (2.59–2.64)	174 (2.26) (1.93–2.6)	306 (2.33) (2.07–2.59)
Primary payer	Medicare	4,871,163 (75.76) (75.72–75.79)	1,163,868 (73.24) (73.17–73.31)	3,722 (46.49) (45.4–47.58)	5,499 (39.98) (39.16–40.8)
	Medicaid	409,845 (6.37) (6.36–6.39)	142,705 (8.98) (8.94–9.02)	2,488 (31.07) (30.06–32.09)	5,027 (36.55) (35.74–37.35)
	Private Insurance	927,617 (14.43) (14.4–14.45)	217,274 (13.67) (13.62–13.73)	1,088 (13.59) (12.84–14.34)	1,514 (11.01) (10.48–11.53)
	Self-pay	110,535 (1.72) (1.71–1.73)	33,877 (2.13) (2.11–2.15)	489 (6.11) (5.59–6.64)	1,189 (8.65) (8.18–9.12)
	No charge	12,560 (0.2) (0.19–0.2)	3,541 (0.22) (0.22–0.23)	34 (0.43) (0.29–0.57)	147 (1.07) (0.9–1.24)
	Other	98,229 (1.53) (1.52–1.54)	27,930 (1.76) (1.74–1.78)	184 (2.3) (1.97–2.63)	377 (2.74) (2.47–3.02)
Median household Income^b^ ($)	<50,000	1,811,558 (28.64) (28.6–28.68)	537,355 (34.42) (34.34–34.49)	2,324 (30.89) (29.85–31.94)	7,567 (56.68) (55.84–57.52)
	50,000–64,999	1,628,021 (25.74) (25.7–25.77)	415,619 (26.62) (26.55–26.69)	1,917 (25.49) (24.51–26.48)	2,806 (21.02) (20.33–21.71)
	65,000–85,999	1,517,440 (23.99) (23.96–24.02)	343,897 (22.03) (21.96–22.09)	1,995 (26.52) (25.53–27.52)	1,952 (14.62) (14.02–15.22)
	>86,000	1,368,203 (21.63) (21.6–21.66)	264,468 (16.94) (16.88–17)	1,286 (17.09) (16.24–17.94)	1,026 (7.69) (7.23–8.14)
US region	Northeast	929,279 (14.44) (14.41–14.46)	320,624 (20.15) (20.09–20.21)	312 (3.9) (3.48–4.32)	2,367 (17.21) (16.58–17.84)
	Midwest	1,387,164 (21.55) (21.52–21.58)	358,497 (22.53) (22.46–22.59)	1,128 (14.07) (13.31–14.84)	2,847 (20.7) (20.03–21.38)
	South	2,245,973 (34.89) (34.86–34.93)	660,944 (41.54) (41.46–41.61)	1,703 (21.25) (20.36–22.15)	6,199 (45.07) (44.24–45.9)
	West	1,874,196 (29.12) (29.08–29.15)	251,225 (15.79) (15.73–15.84)	4,869 (60.77) (59.7–61.84)	2,340 (17.01) (16.38–17.64)
Length of hospital stay (days)	Mean, SD	6, 7.26	9, 9.41	7.64, 8.23	7.61, 9.29
	0–3	2,725,891 (42.35) (42.31–42.39)	410,349 (25.79) (25.72–25.86)	2,855 (35.63) (34.58–36.68)	4,984 (36.24) (35.44–37.05)
	4–6	1,663,115 (25.84) (25.81–25.87)	388,724 (24.43) (24.36–24.5)	1,982 (24.74) (23.79–25.68)	3,265 (23.74) (23.03–24.45)
	7–9	889,557 (13.82) (13.79–13.85)	295,790 (18.59) (18.53–18.65)	1,280 (15.97) (15.17–16.77)	2,135 (15.53) (14.92–16.13)
	10–12	442,432 (6.87) (6.85–6.89)	168,767 (10.61) (10.56–10.65)	611 (7.63) (7.05–8.21)	1,149 (8.36) (7.89–8.82)
	>12	715,310 (11.11) (11.09–11.14)	327,590 (20.59) (20.52–20.65)	1,284 (16.03) (15.22–16.83)	2,219 (16.13) (15.52–16.75)
Died during hospitalization	Died	208,253 (3.24) (3.22–3.25)	52,760 (3.32) (3.29–3.35)	352 (4.39) (3.95–4.84)	261 (1.9) (1.67–2.13)
Comorbidities					
Anemia	Yes	1,210,415 (21.99) (21.96–22.03)	366,319 (26.5) (26.43–26.58)	909 (15.36) (14.45–16.28)	2,377 (20.59) (19.85–21.32)
Arthritis	Yes	191,505 (3.48) (3.46–3.5)	46,113 (3.34) (3.31–3.37)	203 (3.43) (2.97–3.89)	304 (2.63) (2.34–2.92)
Chronic pulmonary disease	Yes	1,736,292 (28.86) (28.82–28.89)	366,101 (24.47) (24.4–24.54)	2,529 (33.54) (32.47–34.61)	4,249 (33.1) (32.28–33.91)
Congestive heart failure	Yes	872,257 (15.85) (15.82–15.88)	238,668 (17.27) (17.2–17.33)	1,535 (25.95) (24.84–27.07)	2,074 (17.96) (17.26–18.66)
Coagulopathy	Yes	310,373 (5.64) (5.62–5.66)	59,516 (4.31) (4.27–4.34)	403 (6.8) (6.16–7.45)	560 (4.85) (4.46–5.24)
Depression	Yes	623,173 (10.36) (10.33–10.38)	137,925 (9.22) (9.17–9.27)	1,058 (14.03) (13.25–14.82)	1,811 (14.1) (13.5–14.71)
Diabetes	Yes	2,547,015 (42.33) (42.29–42.37)	786,192 (52.55) (52.47–52.63)	2,982 (39.54) (38.44–40.65)	5,045 (39.3) (38.45–40.14)
Hypertension	Yes	4,577,849 (76.09) (76.05–76.12)	1,167,531 (78.04) (77.98–78.11)	5,097 (67.59) (66.53–68.65)	9,634 (75.04) (74.29–75.79)
Liver disease	Yes	158,745 (2.88) (2.87–2.9)	30,007 (2.17) (2.15–2.2)	470 (7.94) (7.25–8.63)	971 (8.41) (7.91–8.92)
Obesity	Yes	761,724 (12.66) (12.63–12.69)	159,865 (10.69) (10.64–10.74)	1,246 (16.52) (15.68–17.36)	1,462 (11.39) (10.84–11.94)
Peripheral vascular disorders	Yes	5,047,331 (83.89) (83.86–83.92)	1,027,273 (68.67) (68.59–68.74)	6,878 (91.2) (90.56–91.84)	10,990 (85.61) (85–86.22)
Pulmonary circulation disorders	Yes	168,330 (3.06) (3.04–3.07)	33,916 (2.45) (2.43–2.48)	314 (5.3) (4.73–5.87)	388 (3.36) (3.03–3.69)
Renal failure	Yes	1,571,346 (28.55) (28.51–28.59)	472,719 (34.2) (34.12–34.28)	1,371 (23.17) (22.1–24.25)	3,090 (26.75) (25.95–27.56)

^a^ Admitted patients were identified as non-Hispanic American Indian or Alaska Native, non-Hispanic Asian, non-Hispanic Black, Hispanic, and non-Hispanic White.

^b^ Median household income of residents in the patient's ZIP Code based on 2020.

### Demographic characteristics of methamphetamine and cocaine users

A total of 255,135 (unweighted) patients with methamphetamine use were identified using data from the NIS database 2008–2020. Our study population was predominantly male (58.43% vs. 41.57%), belonged mainly to the age groups 26–40 years (37.17%) and 41–64 years (44.89%), and was predominantly White population (68.8%). A total of 433,584 (unweighted) patients with cocaine use were identified. This cohort was mostly male (60.77% vs. 39.2%), belonged mainly to the age groups 26–40 years (27.2%) and 41–64 years (61.8%), and predominantly Black patients (53.2%), followed by White patients (32.9%). Demographic characteristics of patients diagnosed as methamphetamine and cocaine users are shown in Table 2.

**Table 2:** Demographic characteristics of patients diagnosed as methamphetamine and cocaine users aged 18 or higher from 2008 to 2020 in the US.

Characteristics	Group	Methamphetamine use		Cocaine use	
		Unweighted	Weighted	Unweighted	Weighted
		*n* (%)	*n* (%)	*n* (%)	*n* (%)
		(95% CI)	(95% CI)	(95% CI)	(95% CI)
2008–2020		255,135	1,268,584	433,584	2,133,887
Sex	Men	149,033 (58.43)	741,669 (58.48) (58.39,58.56)	263,420 (60.77) (60.62–60.91)	1,297,598 (60.82) (60.75–60.89)
	Women	106,038 (41.57)	526,602 (41.52) (41.44,41.61)	170,085 (39.23) (39.09–39.38)	835,899 (39.18) (39.11–39.25)
Age (year)	Mean (SD)	42.8, 14.32	42.8, 14.30	46, 12.27	46, 12.28
	18–25	27,767 (10.88)	137,989 (10.88) (10.82,10.93)	27,364 (6.31) (6.24–6.38)	134,411 (6.3) (6.27–6.33)
	26–40	94,823 (37.17)	471,718 (37.18) (37.1,37.27)	117,921 (27.2) (27.06–27.33)	579,568 (27.16) (27.1–27.22)
	41–64	114,533 (44.89)	569,768 (44.91) (44.83,45)	268,224 (61.86) (61.72–62.01)	1,320,409 (61.88) (61.81–61.94)
	≥65	18,012 (7.06)	89,108 (7.02) (6.98,7.07)	20,075 (4.63) (4.57–4.69)	99,498 (4.66) (4.63–4.69)
Race/Ethnicity^a^	White	166,259 (68.84)	826,656 (68.81) (68.72,68.89)	134,573 (32.98) (32.83–33.12)	662,368 (32.93) (32.87–33)
	Black	23,614 (9.78)	117,498 (9.78) (9.73,9.83)	217,268 (53.24) (53.09–53.39)	1,070,674 (53.24) (53.17–53.3)
	Hispanic	34,295 (14.2)	170,858 (14.22) (14.16,14.28)	39,574 (9.7) (9.61–9.79)	195,749 (9.73) (9.69–9.77)
	Asian or Pacific Islander	6,510 (2.7)	32,405 (2.7) (2.67,2.73)	2,286 (0.56) (0.54–0.58)	11,384 (0.57) (0.56–0.58)
	Native American	4,438 (1.84)	22,142 (1.84) (1.82,1.87)	2,004 (0.49) (0.47–0.51)	10,046 (0.5) (0.49–0.51)
	Other	6,403 (2.65)	31,841 (2.65) (2.62,2.68)	12,381 (3.03) (2.98–3.09)	60,977 (3.03) (3.01–3.06)
Primary payer	Medicare	48,658 (19.12)	241,430 (19.08) (19.01–19.15)	98,105 (22.69) (22.57–22.82)	482,666 (22.68) (22.63–22.74)
	Medicaid	123,436 (48.51)	615,274 (48.63) (48.54–48.72)	191,284 (44.25) (44.1–44.39)	943,908 (44.36) (44.29–44.43)
	Private Insurance	34,201 (13.44)	169,454 (13.39) (13.33–13.45)	52,708 (12.19) (12.09–12.29)	259,237 (12.18) (12.14–12.23)
	Self-pay	34,396 (13.52)	170,857 (13.5) (13.44–13.56)	64,038 (14.81) (14.71–14.92)	313,405 (14.73) (14.68–14.78)
	No charge	1,951 (0.77)	9,707 (0.77) (0.75–0.78)	7,582 (1.75) (1.71–1.79)	37,301 (1.75) (1.74–1.77)
	Other	11,806 (4.64)	58,460 (4.62) (4.58–4.66)	18,608 (4.3) (4.24–4.36)	91,264 (4.29) (4.26–4.32)
Median household Income^b^ ($)	<50,000	85,969 (36.8)	428,086 (36.86) (36.78–36.95)	219,198 (53.24) (53.09–53.39)	1,078,432 (53.24) (53.17–53.31)
	50,000–64,999	67,032 (28.69)	333,142 (28.69) (28.61–28.77)	91,219 (22.16) (22.03–22.28)	448,670 (22.15) (22.09–22.21)
	65,000–85,999	51,009 (21.83)	253,126 (21.8) (21.72–21.87)	62,384 (15.15) (15.04–15.26)	306,542 (15.13) (15.08–15.18)
	>86,000	29,611 (12.67)	146,894 (12.65) (12.59–12.71)	38,915 (9.45) (9.36–9.54)	191,851 (9.47) (9.43–9.51)
Length of stay (days)	0–3	126,924 (49.75)	631,001 (49.74) (49.66–49.83)	219,445 (50.61) (50.47–50.76)	1,079,710 (50.6) (50.53–50.67)
	4–6	68,039 (26.67)	338,489 (26.68) (26.61–26.76)	116,204 (26.8) (26.67–26.93)	572,278 (26.82) (26.76–26.88)
	7–9	26,905 (10.55)	133,822 (10.55) (10.5–10.6)	45,500 (10.49) (10.4–10.59)	223,883 (10.49) (10.45–10.53)
	10–12	11,611 (4.55)	57,688 (4.55) (4.51–4.58)	19,501 (4.5) (4.44–4.56)	96,075 (4.5) (4.47–4.53)
	>12	21,646 (8.48)	107,535 (8.48) (8.43–8.53)	32,911 (7.59) (7.51–7.67)	161,827 (7.58) (7.55–7.62)
US region	Northeast	11,872 (4.65)	58,974 (4.65)(4.61,4.69)	108,373 (24.99) (24.87–25.12)	536,285 (25.13) (25.07–25.19)
	Midwest	44,672 (17.51)	221,886 (17.49)(17.42,17.56)	89,885 (20.73) (20.61–20.85)	442,534 (20.74) (20.68–20.79)
	South	69,402 (27.2)	345,324 (27.22)(27.14,27.3)	190,155 (43.86) (43.71–44)	932,452 (43.7) (43.63–43.76)
	West	129,189 (50.64)	642,501 (50.64)(50.55,50.73)	45,171 (10.42) (10.33–10.51)	222,615 (10.43) (10.39–10.47)

^a^ Admitted patients were identified as non-Hispanic American Indian or Alaska Native, non-Hispanic Asian, non-Hispanic Black, Hispanic, and non-Hispanic White.

^b^ Median household income of residents in the patient's ZIP Code based on 2,020.

### Demographic, race/ethnicity, sex-related, and regional disparities of trends in PAD-related hospitalizations from 2008 to 2020

**Figure 2:**
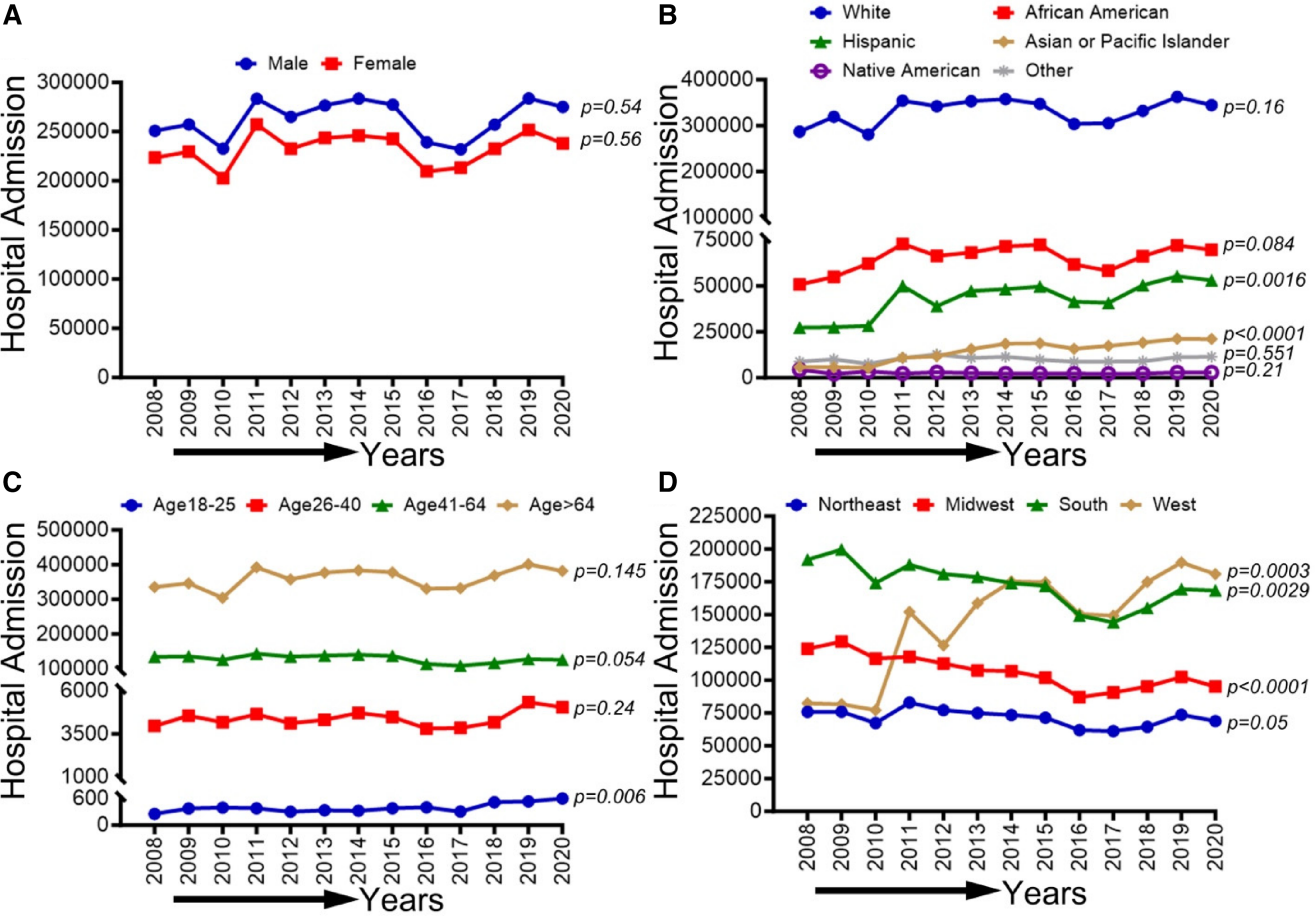
Trends in prevalence of PAD-related hospitalizations from 2008 to 2020 by sex (**A**), race/ethnicity (**B**), age groups (**C**), and region (**D**) in the US. Linear trend test was used to compare the trend from 2008 to 2020 and Cochran-Armitage test was used to compare the group trends. *P < 0.05, **P < 0.01, and ***P < 0.001.

In Figure 2, we observed an increasing trend among male, female, Non-Hispanic White population, Non-Hispanic Black population, and age groups (26–40, 41–64, and ≥65 years old) that did not achieve statistical significance (*p*-trend >0.05). On the other hand, a statistically significant increasing trend in PAD-related hospitalization was observed in Hispanics (*p*-trend <0.001), Asian or Pacific Islanders (*p*-trend <0.001), patients 18–25 years old (*p*-trend <0.05), the western region (*p*-trend <0.001). At the same time, a decreasing trend in PAD-related hospitalization was observed in the midwestern region (*p*-trend <0.001) and the southern region (*p*-trend <0.01). Trends in the prevalence of PAD-related hospitalizations and in-hospital mortality from 2008 to 2020 by sex (A), race/ethnicity (B), age groups (C), region (D), and all-cause mortality in the US are shown in Figure 2.

### Demographic, race/ethnicity, sex-related, and regional disparities of trends in CLI-related hospitalizations from 2008 to 2020

**Figure 3:**
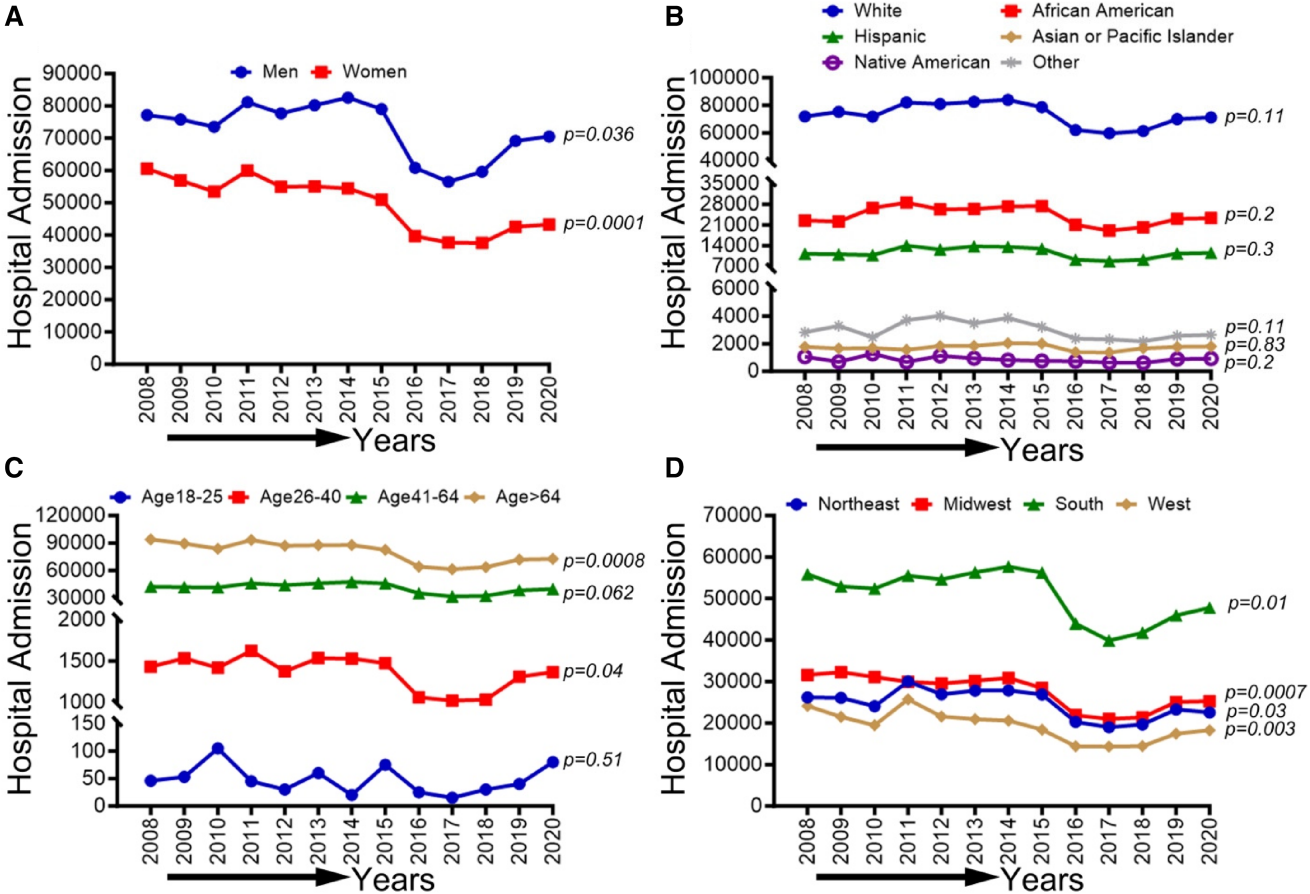
Trends in prevalence of CLI-related hospitalizations from 2008 to 2020 by sex (**A**), race/ethnicity (**B**), age groups (**C**), and region (**D**) in the US. Linear trend test was used to compare the trend from 2008 to 2020 and Cochran-Armitage test was used to compare the group trends. *P < 0.05, **P < 0.01, and ***P < 0.001.

In Figure 3, we observed an overall decreasing trend in CLI-related hospitalization among men (*p*-trend <0.001), women (*p*-trend: 0.036), and patients ≥65 years old (*p*-trend <0.001), and in the northeastern (*P*-trend <0.05), midwestern (*p*-trend <0.001), southern (*p*-trend <0.01), and western regions (*p*-trend <0.001). At the same time, an overall increasing trend was seen for the age group 26–40 years (*p* < 0.05). Trends in the prevalence of CLI-related hospitalizations from 2008 to 2020 by sex (A), race/ethnicity (B), age groups (C), region (D), and all-cause mortality in the US are shown in Figure 3.

### Demographic, race/ethnicity, sex-related, and regional disparities of trends in amputations among CLI-related hospitalizations in PAD patients from 2008 to 2020

**Figure 4:**
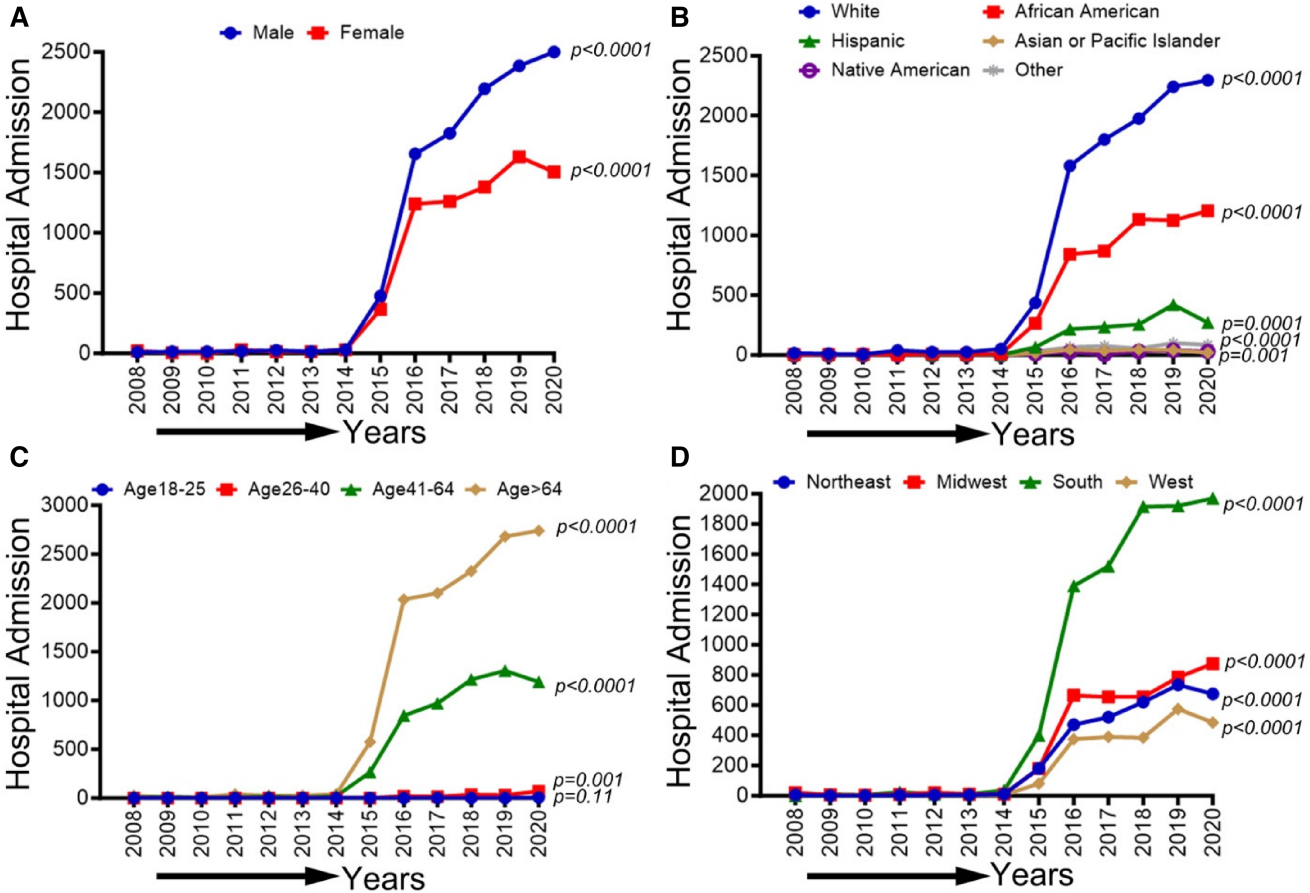
Trends in prevalence of amputations among CLI-related hospitalization patients from 2008 to 2020 by sex (**A**), race/ethnicity (**B**), age groups (**C**), and region in the US. Linear trend test was used to compare the trend from 2008 to 2020 and Cochran-Armitage test was used to compare the group trends. *P < 0.05, **P < 0.01, and ***P < 0.001.

In Figure 4, We observed an increasing trend in CLI-related amputations in hospitalized patients from 2008 to 2020 for men, women, White, African American, Asian or Pacific islanders, Hispanic, and Native Americans (*p*-trend <0.001). Similarly, CLI-related amputations significantly increased in all age groups except age 18–25 years (*p*-trend <0.05) and in the northeastern, midwestern, western, and southern regions (*p*-trend <0.001). Trends in the prevalence of CLI-related amputations among the US population from 2008 to 2020 by sex (A), race/ethnicity (B), age groups (C), and region (D) are shown in Figure 4.

### Demographic, race/ethnicity, sex-related, and regional disparities of trends in PAD-related hospitalizations among methamphetamine users from 2008 to 2020

**Figure 5:**
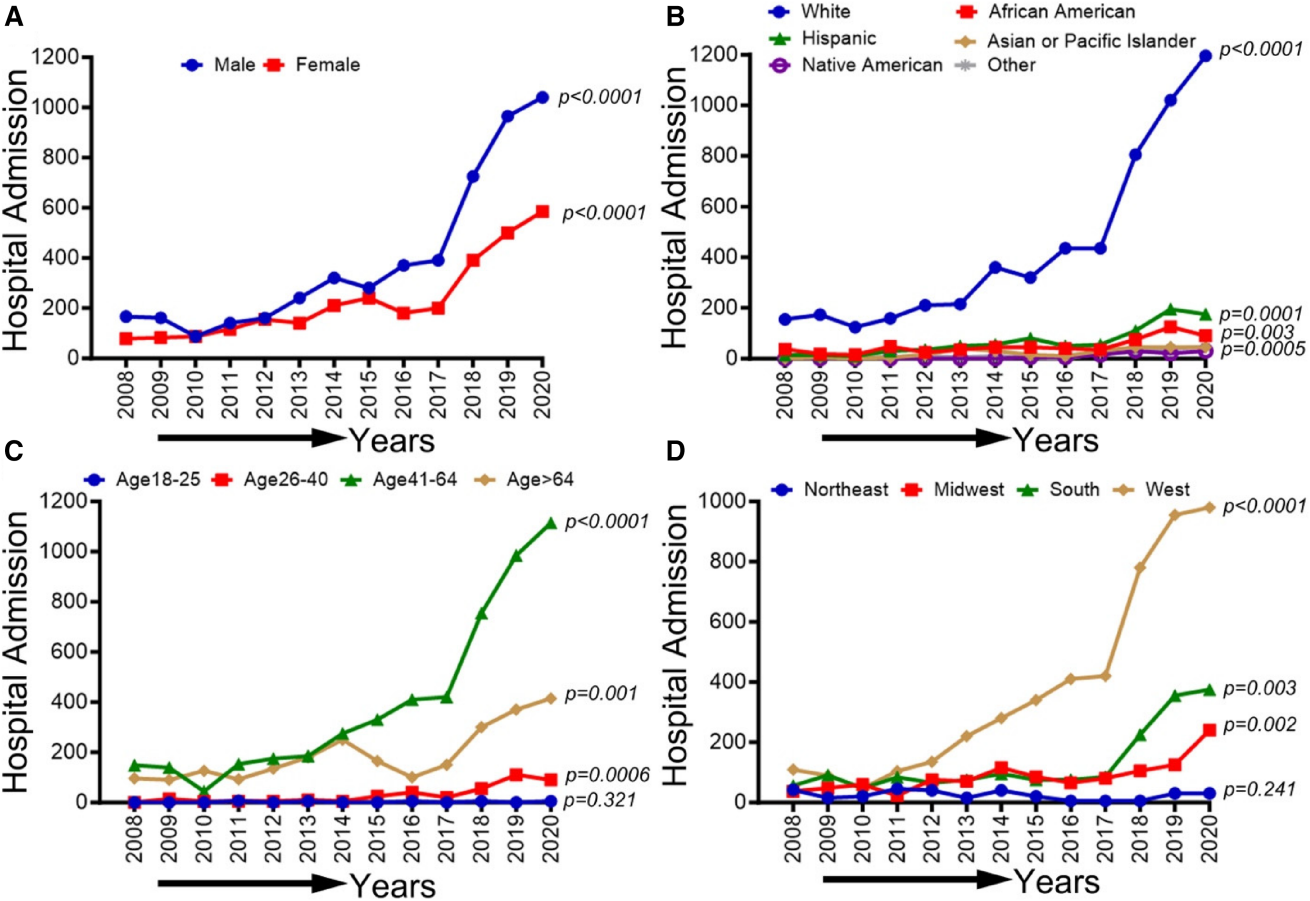
Trends in prevalence of PAD-related hospitalizations among methamphetamine users from 2008 to 2020 by sex (**A**), race/ethnicity (**B**), age groups (**C**), and region (**D**) in the US. Linear trend test was used to compare the trend from 2008 to 2020 and Cochran-Armitage test was used to compare the group trends. *P < 0.05, **P < 0.01, and ***P < 0.001.

In Figure 5, an overall increasing trend was observed in PAD-related hospitalization among methamphetamine users in men and women (*p*-trend <0.05), although it was more remarkable for men than women. An overall increasing trend was observed in PAD-related hospitalization among methamphetamine users for all ethnic and racial groups (*p*-trend <0.05). However, the upward trend was more pronounced for the White patient population. Similarly, an upward trend in hospitalizations was seen for the age groups 26–40, 41–64, and >64 years (*p*-trend <0.05), and the midwestern, southern, and western regions (*p*-trend <0.05), with an upward spike noticed in the age group 41–64 years and the western region, particularly during the study period 2017–2020. Trends in PAD-related hospitalizations among methamphetamine users (2008–2020) by sex (A), race/ethnicity (B), age groups (C), and region (D) are shown in Figure 5.

### Demographic, race/ethnicity, sex-related, and regional disparities of trends in PAD-related hospitalizations among cocaine users from 2008 to 2020

**Figure 6:**
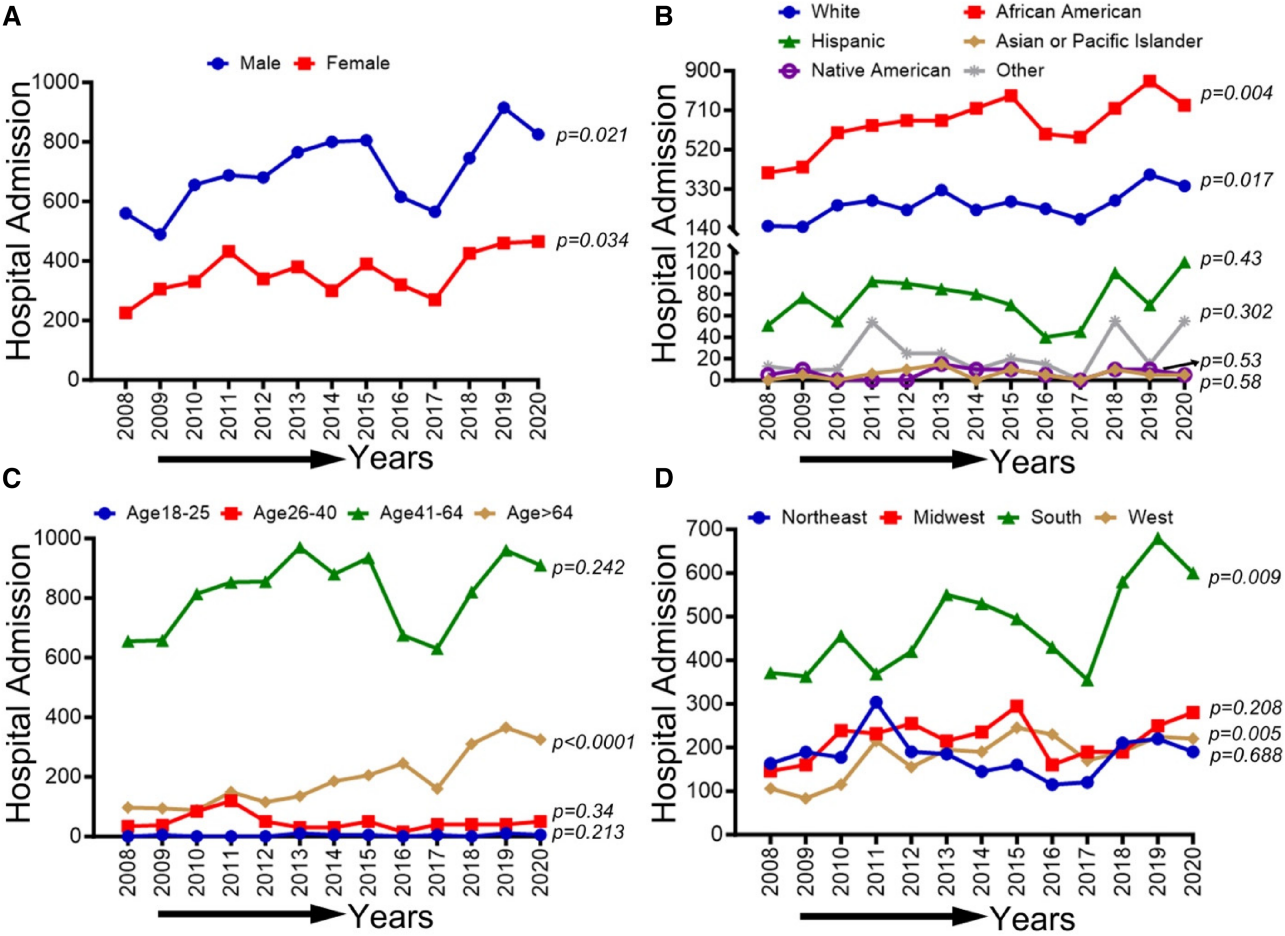
Trends in prevalence of PAD-related hospitalizations among cocaine users from 2008 to 2020 by sex (**A**), race/ethnicity (**B**), age groups (**C**), and region (**D**) in the US. Linear trend test was used to compare the trend from 2008 to 2020 and Cochran-Armitage test was used to compare the group trends. *P < 0.05, **P < 0.01, and ***P < 0.001.

In Figure 6, an overall increasing trend was observed in PAD-related hospitalization among cocaine users for men and women (*p*-trend <0.05). A similar uptrend was observed for both Black/African American and White patient populations (*p*-trend <0.05), but the upward trend was more pronounced in African Americans. No significant change in the overall trend was observed in other ethnic/racial groups (*p*-trend >0.05). Furthermore, an upward trend in hospitalizations was seen for the age group >64 years (*p*-trend <0.05) and in the southern and western regions (*p*-trend <0.05). However, these trends did not change significantly for other age groups and US regions (*p*-trend >0.05). Trends in PAD-related hospitalizations among cocaine users (2008–2020) by sex (A), race/ethnicity (B), age groups (C), and region (D) are shown in Figure 6.

### Demographic, race/ethnicity, sex-related, and regional disparities of trends in CLI-related hospitalizations among methamphetamine users from 2008 to 2020

**Figure 7:**
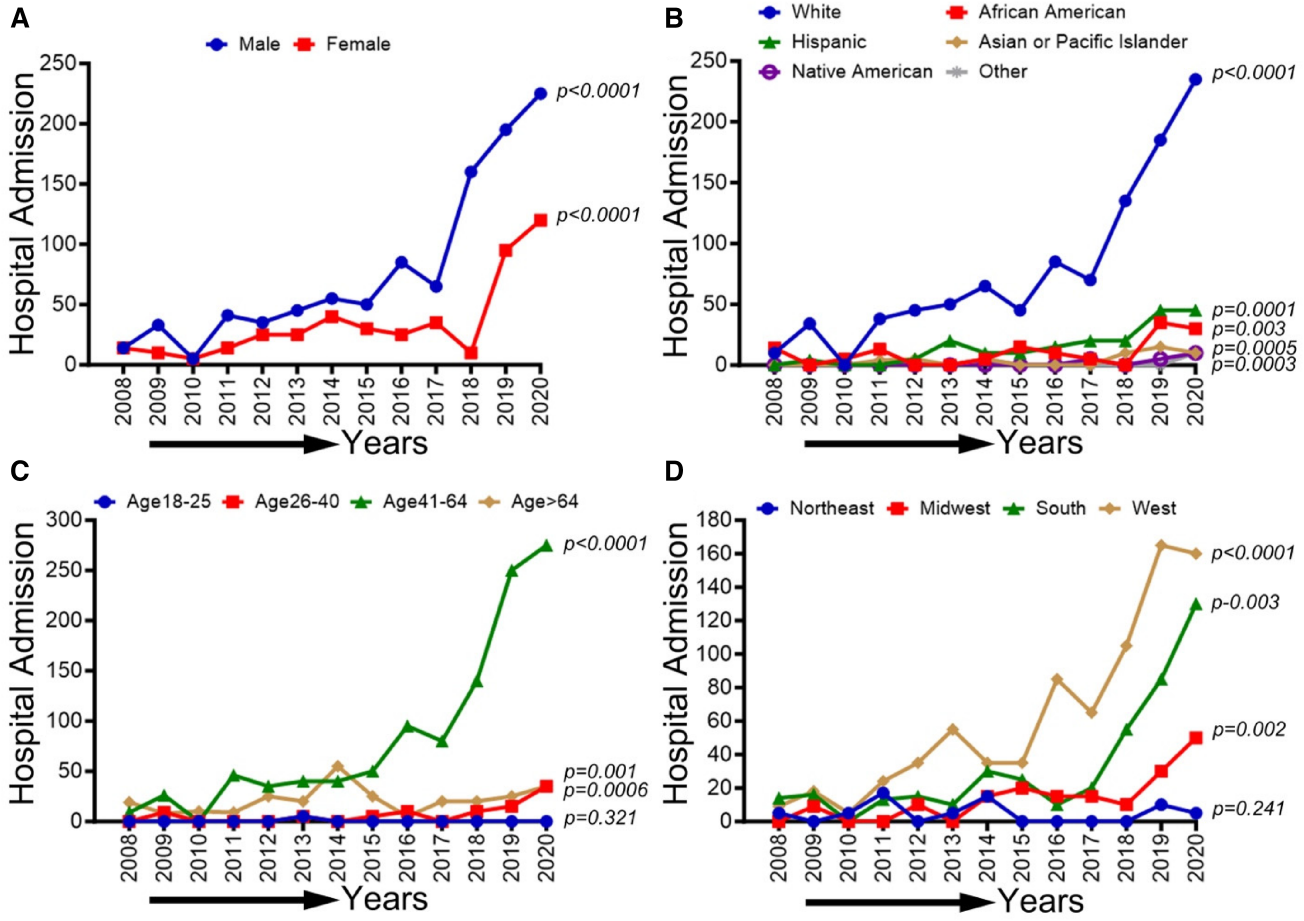
Trends in prevalence of CLI-related hospitalizations among methamphetamine users from 2008 to 2020 by sex (**A**), race/ethnicity (**B**), age groups (**C**), and region (**D**) in the US. Linear trend test was used to compare the trend from 2008 to 2020 and Cochran-Armitage test was used to compare the group trends. *P < 0.05, **P < 0.01, and ***P < 0.001.

In Figure 7, we observed an overall increasing trend in CLI-related hospitalization among methamphetamine users for both males and females from 2008 to 2017, which showed a concerning rapid upward spike from 2018 to 2020 (*p*-trend <0.01). A similar upward trend in CLI-related hospitalization in methamphetamine users was observed for the White population (*p*-trend <0.01), African Americans (*p*-trend <0.01), Hispanics (*p*-trend <0.01), Asian/PI (*p*-trend <0.01), Native Americans (*p*-trend <0.05) and population belong to age groups 26–40, 41–64 & >64 years (*p*-trend <0.01). Geographically, we observed an increasing trend in the midwestern (*p*-trend <0.01), the southern (*p*-trend <0.01), and the western regions (*p*-trend <0.01). An upward change, particularly after the study period 2017, was noticed in these trends. Trends in CLI-related hospitalizations among methamphetamine users (2008–2020) by sex (A), race/ethnicity (B), age groups (C), and region (D) are shown in Figure 7.

### Demographic, race/ethnicity, sex-related, and regional disparities of trends in CLI-related hospitalizations among cocaine users from 2008 to 2020

**Figure 8:**
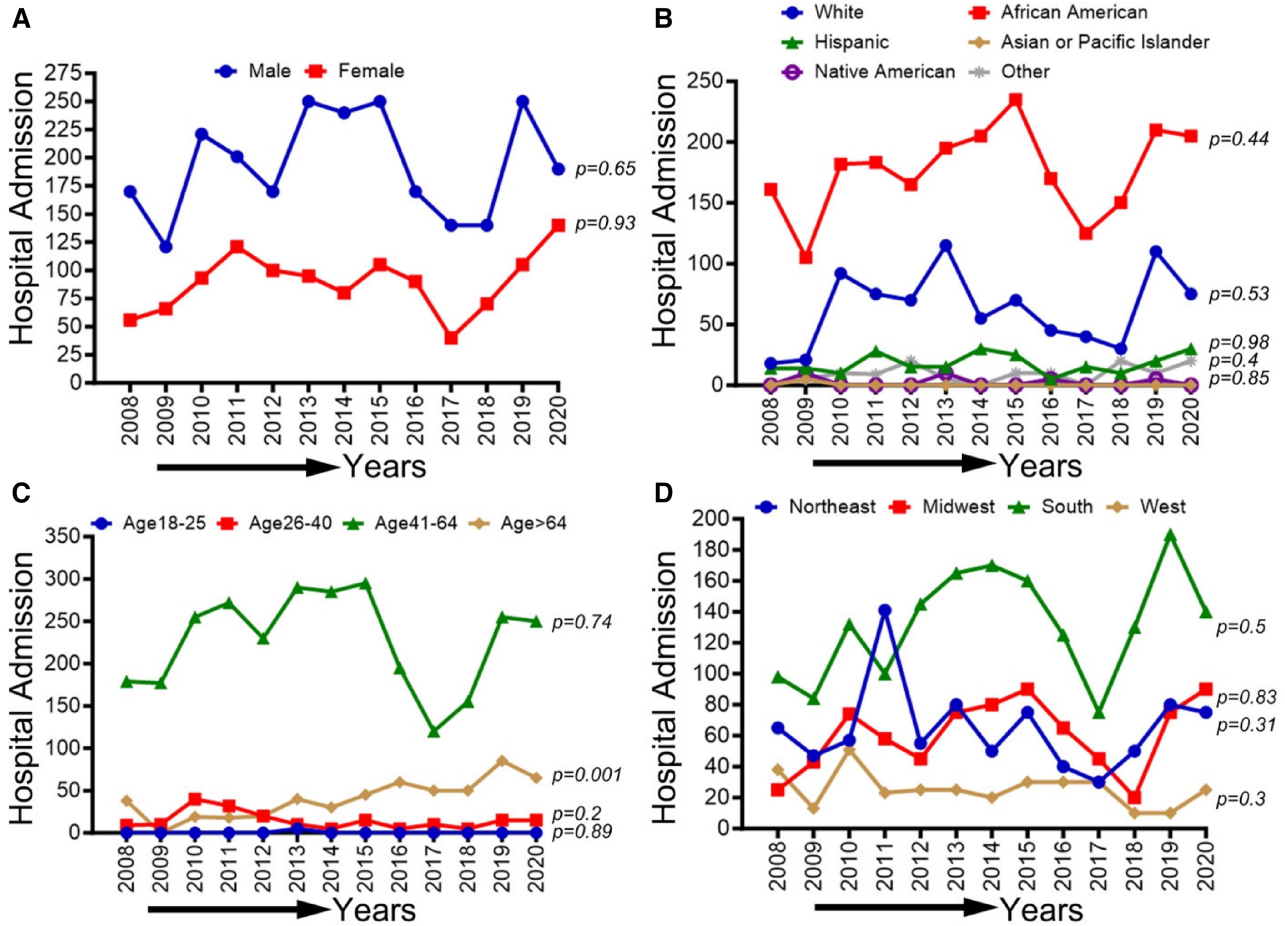
Trends in prevalence of CLI-related hospitalizations among cocaine users from 2008 to 2020 by sex (**A**), race/ethnicity (**B**), Age groups (**C**), and region (**D**) in the US. Linear trend test was used to compare the trend from 2008 to 2020 and Cochran-Armitage test was used to compare the group trends. *P < 0.05, **P < 0.01, and ***P < 0.001.

CLI-related hospitalization among cocaine users in patients ≥65 years old showed an overall increasing trend (*p*-trend <0.01). The other trends based on sex, ethnic/racial groups, and regions did not achieve statistical significance (*p*-trend >0.05). Trends in CLI-related hospitalizations among cocaine users (2008–2020) by sex (A), race/ethnicity (B), age groups (C), and region (D) are shown in Figure 8.

### The unadjusted and adjusted odds ratios for PAD and CLI among methamphetamine users from 2008 to 2020 in the adult population in the US

In Table 3, among methamphetamine users, there was no significant difference in the adjusted odds of PAD and CLI for women compared to men (aOR 0.79, 95% CI 0.6–1.04). Older age was significantly associated with higher adjusted odds of PAD and CLI among methamphetamine users; compared to the age group 18–25 years, patients in the age group 41–64 years had higher odds of PAD (aOR 5.57, 95% CI 3.1–11) and CLI (aOR 10.69, 95% CI 2.35–189). Similarly, patients in the age group ≥65 years had higher adjusted odds of PAD (aOR 8.18, 95% CI 4.46- 16.8) and CLI (aOR 10.2, 95% CI 2.12–183.5) compared to the patients in the age group 18–25 years. Methamphetamine-related hospitalizations from the western region had significantly higher adjusted odds of PAD (aOR 1.93, 95% CI 1.48–2.55) compared to those in the northeastern region, while there were no other significant regional differences for the adjusted odds of CLI in this cohort. Among various comorbidities identified in the methamphetamine-related hospitalizations, we observed higher adjusted odds of PAD in patients with arthritis (aOR 1.43, 95% CI 1.02–1.98), PAD (aOR 1.43, 95% CI 1.26–1.62), and CLI (aOR 2.69, 95% CI 2.07–3.5) in diabetics and PAD (aOR 1.16, 95% CI 1.02–1.31) in patients with hypertension. The unadjusted and adjusted odds ratios for PAD and CLI among methamphetamine users from 2008 to 2020 in the adult population in the US are presented in Table 3.

**Table 3:** Unadjusted and adjusted odds ratio for PAD and CLI among methamphetamine users from 2,008–2,020 in the US.

		PAD patients with meth use		CLI patients with meth use	
		Unadjusted odds ratio (95% CI)	Adjusted odds ratio (95% CI)	Unadjusted odds ratio (95% CI)	Adjusted odds ratio (95% CI)
Gender	Men	[Reference]	[Reference]	[Reference]	[Reference]
	Women	0.82 (0.75, 0.89)	1.1 (0.97, 1.24)	0.64 (0.52, 0.78)	0.79 (0.6, 1.04)
Age (year)	18–25	[Reference]	[Reference]	[Reference]	[Reference]
	26–40	3.21 (1.81, 6.31)	1.45 (0.78, 3.02)	8.39 (1.8, 149.43)	2.66 (0.53, 48.15)
	41–64	35.56 (20.71, 68.61)	5.57 (3.1, 11.3)	79.78 (18.04, 1,401.1)	10.69 (2.35, 189.19)
	≥65	127.96 (74.26, 247.47)	8.18 (4.46, 16.83)	164.92 (36.68, 2,909.27)	10.2 (2.12, 183.51)
Race/Ethnicity^a^	White patients	[Reference]	[Reference]	[Reference]	[Reference]
	Black patients	0.83 (0.72, 0.96)	0.88 (0.72, 1.07)	0.94 (0.67, 1.29)	0.69 (0.44, 1.04)
	Hispanic patients	0.81 (0.71, 0.93)	0.9 (0.74, 1.08)	0.86 (0.63, 1.15)	0.68 (0.45, 1.02)
	Asian or Pacific Islander patients	1.37 (1.07, 1.74)	1.01 (0.72, 1.4)	1.33 (0.7, 2.26)	0.95 (0.45, 1.79)
	Native American patients	0.7 (0.48, 0.97)	1.03 (0.61, 1.64)	0.7 (0.27, 1.43)	0.93 (0.28, 2.28)
	Other patients	0.76 (0.56, 1)	0.81 (0.52, 1.22)	0.54 (0.21, 1.11)	0.44 (0.11, 1.2)
Primary payer	Medicare	[Reference]	[Reference]	[Reference]	[Reference]
	Medicaid	0.26 (0.24, 0.29)	0.76 (0.65, 0.9)	0.41 ( 0.33, 0.51)	1.26 (0.91, 1.76)
	Private insurance	0.37 (0.32, 0.42)	1.12 (0.92, 1.36)	0.27 (0.18, 0.39)	0.66 (0.37, 1.09)
	Self-pay	0.17 (0.14, 0.2)	1.02 (0.79, 1.32)	0.23 (0.15, 0.33)	1.18 (0.67, 2)
	No charge	0.29 (0.17, 0.47)	2.15 (1.08, 4.02)	0.38 (0.09, 0.99)	1.67 (0.26, 5.74)
	Other	0.23 (0.17, 0.29)	0.89 (0.63, 1.23)	0.31 (0.17, 0.51)	1.13 (0.54, 2.15)
Median household income^b^ ($)	<50,000	[Reference]	[Reference]	[Reference]	[Reference]
	50,000–64,999	1.05 (0.93, 1.17)	0.88 (0.76, 1.03)	0.88 (0.69, 1.13)	0.81 (0.59, 1.11)
	65,000–85,999	1.38 (1.24, 1.55)	1.07 (0.92, 1.26)	0.87 (0.67, 1.14)	0.8 (0.57, 1.13)
	>86,000	1.44 (1.26, 1.64)	1.04 (0.86, 1.25)	0.67 (0.46, 0.95)	0.67 (0.43, 1.01)
US region	Northeast	[Reference]	[Reference]	[Reference]	[Reference]
	Midwest	1.05 (0.83, 1.34)	1.05 (0.78, 1.44)	0.83 (0.49, 1.5)	0.54 (0.28, 1.08)
	South	1.1 (0.88, 1.4)	1.26 (0.95, 1.69)	1.42 (0.88, 2.45)	0.92 (0.52, 1.73)
	West	1.91 (1.55, 2.39)	1.93 (1.48, 2.55)	1.59 (1.01, 2.71)	1.04 (0.61, 1.9)
Length of hospital stay (days)	0–3	[Reference]	[Reference]	[Reference]	[Reference]
	4–6	1.28 (1.15, 1.43)	1.06 (0.91, 1.22)	2.86 (2.08, 3.97)	2.03 (1.37, 3.04)
	7–9	1.92 (1.69, 2.17)	1.4 (1.17, 1.67)	6.37 (4.58, 8.9)	4.35 (2.91, 6.56)
	10–12	2.35 (1.99, 2.75)	1.49 (1.19, 1.87)	8.89 (6.1, 12.91)	4.63 (2.81, 7.5)
	>12	2.45 (2.16, 2.77)	1.32 (1.1, 1.59)	11.91 (8.81, 16.29)	6.86 (4.71, 10.11)
Anemia	No	[Reference]	[Reference]	[Reference]	[Reference]
	Yes	2.12 (1.86, 2.42)	1.17 (0.99, 1.39)	2.58 (1.9, 3.43)	1.23 (0.87, 1.72)
Arthritis	No	[Reference]	[Reference]	[Reference]	[Reference]
	Yes	2.48 (1.87, 3.22)	1.43 (1.02, 1.98)	1.78 (0.76, 3.49)	1.45 (0.6, 2.97)
Chronic pulmonary disease	No	[Reference]	[Reference]	[Reference]	[Reference]
	Yes	2.36 (2.16, 2.58)	0.99 (0.88, 1.12)	2.03 (1.64, 2.5)	0.88 (0.66, 1.16)
Congestive heart failure	No	[Reference]	[Reference]	[Reference]	[Reference]
	Yes	3.37 (3.01, 3.77)	0.62 (0.54, 0.72)	4.58 (3.56, 5.84)	1.17 (0.87, 1.57)
Coagulopathy	No	[Reference]	[Reference]	[Reference]	[Reference]
	Yes	2.02 (1.68, 2.41)	0.92 (0.73, 1.15)	1.87 (1.16, 2.84)	0.6 (0.34, 0.99)
Depression	No	[Reference]	[Reference]	[Reference]	[Reference]
	Yes	0.92 (0.82, 1.04)	0.98 (0.83, 1.15)	0.71 (0.52, 0.95)	0.89 (0.61, 1.26)
Diabetes	No	[Reference]	[Reference]	[Reference]	[Reference]
	Yes	3.49 (3.2, 3.81)	1.43 (1.26, 1.62)	6.48 (5.32, 7.9)	2.69 (2.07, 3.5)
Hypertension	No	[Reference]	[Reference]	[Reference]	[Reference]
	Yes	4.6 (4.2, 5.03)	1.16 (1.02, 1.31)	4.56 (3.71, 5.64)	1.13 (0.86, 1.51)
Liver disease	No	[Reference]	[Reference]	[Reference]	[Reference]
	Yes	1.36 (1.15, 1.6)	0.98 (0.8, 1.2)	1.37 (0.9, 2)	0.94 (0.59, 1.42)
Obesity	No	[Reference]	[Reference]	[Reference]	[Reference]
	Yes	1.69 (1.5, 1.9)	1.04 (0.88, 1.23)	1.3 (0.94, 1.74)	0.77 (0.52, 1.11)
Peripheral vascular disorders	No	[Reference]	[Reference]	[Reference]	[Reference]
	Yes	503.71 (436.06, 585.11)	259.05 (219.12, 308.13)	175 (137.15, 226.12)	64.99 (48.39, 88.41)
Pulmonary circulation disorders	No	[Reference]	[Reference]	[Reference]	[Reference]
	Yes	2.85 (2.27, 3.52)	0.92 (0.7, 1.19)	2.26 (1.2, 3.85)	0.57 (0.29, 1.03)
Renal failure	No	[Reference]	[Reference]	[Reference]	[Reference]
	Yes	4.77 (4.24, 5.35)	1.15 (0.98, 1.34)	6.3 (4.85, 8.12)	1.65 (1.2, 2.24)

^a^ Admitted patients were identified as non-Hispanic American Indian or Alaska Native people, non-Hispanic Asian people, non-Hispanic Black people, Hispanic people, and non-Hispanic White people.

^b^ Median household income of residents in the patient's ZIP Code based on 2020.

### Unadjusted and adjusted odds ratios for PAD and CLI among cocaine users from 2008 to 2020 in the adult population in the US

In Table 4, we observed women had significantly higher adjusted odds of CLI (aOR 1.2, 95% CI 1.1–1.3) compared to men, while there was no significant difference for PAD (aOR 0.94, 95% CI 0.8–1.09) among patients hospitalized with cocaine use. Older age was significantly associated with higher adjusted odds of PAD and CLI among cocaine users; compared to the age group 18–25 years, patients in the age group 26–40 years had higher adjusted odds of PAD (aOR 3.49, 95% CI 1.07–21.4) and CLI (aOR 2.4, 95% CI 1.36–4.73). Similarly, patients with cocaine use belonging to the age groups 41–64 and ≥65 years had higher adjusted odds of PAD (aOR 11.7, 95% CI 3.7–71 and aOR 14.3, 95% CI 4.4–87 respectively) and CLI (aOR 7, 95% CI 4–13.7 and aOR 9.7, 95% CI 5.5–18.97 respectively) when compared to patients in the age group 18–25 years. Non-Hispanic Blacks hospitalized with cocaine use had significantly higher adjusted odds of CLI compared to Non-Hispanic Whites (aOR 1.1, 95% CI 1.01–1.21), while there was no significant difference when comparing the other ethnic/racial groups. Similarly, cocaine-related hospitalizations from the southern (aOR 1.22, 95% CI 1.11–1.36) and western regions (2.35, 95% CI 2.08–2.67) had significantly higher adjusted odds of CLI compared to the northeastern region. In all age groups, among various comorbidities identified in this cohort, diabetes had a higher adjusted odds of PAD (aOR 1.85, 95% CI 1.61–2.12) and CLI (aOR 1.36, 95% CI 1.25–1.47). Similarly, in all age groups, hypertension was associated with higher adjusted odds of PAD (aOR 1.4, 95% CI 1.2- 1.65) and CLI (aOR 1.31, 95% CI 1.2–1.43). The unadjusted and adjusted odds ratios for PAD and CLI among cocaine users from 2008 to 2020 in the US adult population are presented in Table 4.

**Table 4:** Unadjusted and adjusted odds ratio for PAD and CLI among cocaine users from 2008–2020 in the US.

		PAD patients with cocaine use		CLI patients with cocaine use	
		Unadjusted odds ratio (95% CI)	Adjusted odds ratio (95% CI)	Unadjusted odds ratio (95% CI)	Adjusted odds ratio (95% CI)
Gender	Male	[Reference]	[Reference]	[Reference]	[Reference]
	Female	0.69 (0.61, 0.78)	0.94 (0.8, 1.09)	0.79 (0.74, 0.84)	1.2 (1.1, 1.3)
Age (year)	18–25	[Reference]	[Reference]	[Reference]	[Reference]
	26–40	6.77 (2.12, 41.33)	3.49 (1.07, 21.42)	3.33 (2.04, 5.87)	2.4 (1.36, 4.73)
	41–64	58.79 (19.01, 353.98)	11.75 (3.76, 71.09)	28.11 (17.61, 48.9)	7.07 (4.08, 13.75)
	≥65	175.64 (56.28, 1,061.18)	14.29 (4.47, 87.23)	105.97 (66.08, 184.88)	9.68 (5.51, 18.97)
Race/Ethnicity^a^	White patients	[Reference]	[Reference]	[Reference]	[Reference]
	Black patients	2.22 (1.94, 2.54)	1.14 (0.97, 1.34)	1.83 (1.72, 1.96)	1.1 (1.01, 1.21)
	Hispanic patients	1.23 (0.97, 1.54)	0.92 (0.69, 1.2)	1.09 (0.96, 1.22)	0.99 (0.85, 1.15)
	Asian or Pacific Islander patients	0.51 (0.08, 1.59)	0 (0, 0)	1.38 (0.89, 2.03)	0.89 (0.49, 1.52)
	Native American patients	2.15 (1.03, 3.93)	1.73 (0.76, 3.43)	1.34 (0.87, 1.97)	1.02 (0.6, 1.67)
	Other patients	1.46 (1.02, 2.02)	1.29 (0.86, 1.89)	1 (0.81, 1.21)	1.12 (0.87, 1.43)
Primary payer	Medicare	[Reference]	[Reference]	[Reference]	[Reference]
	Medicaid	0.48 (0.42, 0.54)	1 (0.85, 1.18)	0.43 (0.4, 0.46)	0.91 (0.83, 1)
	Private insurance	0.33 (0.26, 0.4)	0.85 (0.66, 1.08)	0.43 (0.39, 0.48)	1.13 (1, 1.29)
	Self-pay	0.25 (0.2, 0.31)	0.77 (0.58, 1.01)	0.29 (0.26, 0.32)	1.03 (0.89, 1.19)
	No charge	0.2 (0.1, 0.37)	0.55 (0.23, 1.1)	0.32 (0.24, 0.41)	1.11 (0.79, 1.54)
	Other	0.34 (0.24, 0.46)	0.79 (0.53, 1.15)	0.34 (0.28, 0.4)	0.82 (0.66, 1.01)
Median household Income^b^ ($)	<50,000	[Reference]	[Reference]	[Reference]	[Reference]
	50,000–64,999	0.82 (0.71, 0.95)	1.03 (0.87, 1.22)	0.88 (0.82, 0.95)	0.99 (0.9, 1.08)
	65,000–85,999	0.71 (0.6, 0.84)	0.93 (0.76, 1.14)	0.82 (0.75, 0.89)	0.97 (0.87, 1.08)
	>86,000	0.51 (0.4, 0.63)	0.78 (0.59, 1.02)	0.65 (0.58, 0.72)	0.93 (0.81, 1.08)
US region	Northeast	[Reference]	[Reference]	[Reference]	[Reference]
	Midwest	1.24 (1.05, 1.46)	0.83 (0.68, 1.01)	1.75 (1.6, 1.92)	1.09 (0.97, 1.22)
	South	1.39 (1.21, 1.6)	1.06 (0.9, 1.26)	1.86 (1.71, 2.02)	1.22 (1.11, 1.36)
	West	1.25 (1.02, 1.53)	0.96 (0.75, 1.22)	2.96 (2.69, 3.26)	2.35 (2.08, 2.67)
Length of hospital stay (days)	0–3	[Reference]	[Reference]	[Reference]	[Reference]
	4–6	2.17 (1.82, 2.59)	2 (1.63, 2.44)	1.21 (1.12, 1.3)	1.12 (1.01, 1.23)
	7–9	4.66 (3.88, 5.6)	3.6 (2.91, 4.45)	2.01 (1.84, 2.19)	1.57 (1.4, 1.76)
	10–12	6.91 (5.61, 8.49)	4.76 (3.72, 6.05)	2.58 (2.31, 2.88)	1.68 (1.45, 1.94)
	>12	9.08 (7.69, 10.73)	6.55 (5.39, 7.98)	2.72 (2.49, 2.96)	1.87 (1.67, 2.09)
Anemia	No	[Reference]	[Reference]	[Reference]	[Reference]
	Yes	2.78 (2.42, 3.19)	1.37 (1.17, 1.61)	2.17 (2.01, 2.35)	1.12 (1.02, 1.23)
Arthritis	No	[Reference]	[Reference]	[Reference]	[Reference]
	Yes	1.83 (1.19, 2.68)	1.01 (0.62, 1.54)	1.97 (1.6, 2.4)	1.03 (0.81, 1.3)
Chronic pulmonary disease	No	[Reference]	[Reference]	[Reference]	[Reference]
	Yes	1.35 (1.19, 1.53)	0.79 (0.68, 0.91)	1.78 (1.67, 1.9)	0.97 (0.9, 1.06)
Congestive heart failure	No	[Reference]	[Reference]	[Reference]	[Reference]
	Yes	3.07 (2.58, 3.62)	0.81 (0.67, 0.98)	3.38 (3.1, 3.68)	0.75 (0.68, 0.84)
Coagulopathy	No	[Reference]	[Reference]	[Reference]	[Reference]
	Yes	1.25 (0.92, 1.66)	0.6 (0.43, 0.81)	1.62 (1.41, 1.85)	0.82 (0.69, 0.96)
Depression	No	[Reference]	[Reference]	[Reference]	[Reference]
	Yes	1.01 (0.86, 1.19)	0.96 (0.8, 1.16)	1.01 (0.92, 1.09)	0.97 (0.87, 1.07)
Diabetes	No	[Reference]	[Reference]	[Reference]	[Reference]
	Yes	4.4 (3.92, 4.94)	1.85 (1.61, 2.12)	3.27 (3.07, 3.48)	1.36 (1.25, 1.47)
Hypertension	No	[Reference]	[Reference]	[Reference]	[Reference]
	Yes	4.36 (3.83, 4.97)	1.4 (1.2, 1.65)	4.38 (4.1, 4.69)	1.31 (1.2, 1.43)
Liver disease	No	[Reference]	[Reference]	[Reference]	[Reference]
	Yes	1.57 (1.29, 1.89)	1.13 (0.91, 1.39)	1.56 (1.41, 1.73)	1.12 (0.99, 1.26)
Obesity	No	[Reference]	[Reference]	[Reference]	[Reference]
	Yes	1.15 (0.94, 1.39)	0.78 (0.61, 0.97)	1.29 (1.16, 1.42)	0.84 (0.74, 0.95)
Peripheral vascular disorders	No	[Reference]	[Reference]	[Reference]	[Reference]
	Yes	110.7 (97.63, 125.79)	59.72 (51.5, 69.4)	323.54 (297, 353.03)	217.5 (197.1, 240.45)
Pulmonary circulation disorders	No	0 (0, 0)	[Reference]	[Reference]	[Reference]
	Yes	1.9 (1.21, 2.82)	0.66 (0.41, 1)	2.98 (2.48, 3.55)	0.93 (0.75, 1.15)
Renal failure	No	[Reference]	[Reference]	[Reference]	[Reference]
	Yes	4.27 (3.71, 4.9)	1.02 (0.86, 1.21)	3.96 (3.67, 4.27)	0.95 (0.86, 1.04)

^a^ Admitted patients were identified as non-Hispanic American Indian or Alaska Native people, non-Hispanic Asian people, non-Hispanic Black people, Hispanic people, and non-Hispanic White people.

^b^ Median household income of residents in the patient's ZIP Code based on 2020.

## Discussion

PAD is a disease with an increasingly global reach that has risen in incidence every year since 1990. In the US, 8.5 million individuals (7.2% of the overall population) were affected by PAD in 2000 (5). In our study, we comprehensively investigated the prevalence and trends of PAD from 2008 to 2020. Our study showed a moderate increase in PAD prevalence with a consistently increased male-to-female ratio over the last decade. The geographical distribution of PAD in the US has dramatically changed. Specifically, the western region exhibited a more than twofold rise in PAD in 2020 compared to other areas of the country. Within this, Pacific Islanders and Hispanics showed a striking increase ranging from 2 to 3.6 times in PAD cases compared to other races and ethnicities. However, CLI remained relatively consistent overall. Notably, we observed a substantial surge in PAD cases among methamphetamine users during the study period, particularly in the western region.

Previous studies have shown that the prevalence of PAD increased by 13.1% in high-income countries and more than doubled (28.7%) in low/middle-income countries between 2000 and 2010 (5, 9). In the year 2000, US population-based estimates suggest that over 8 million people aged 40 years and older have been affected by PAD (3). However, some studies showed that PAD as a primary discharge diagnosis declined between 2006 and 2016 (21), we provide data revealing temporal trends in PAD prevalence between 2008 and 2020. Conversely, while the incidence of CLI remained steady between 2003 and 2011 (22), our analysis of the NIS data showed a decrease in the first half of the last decade before it began to rise again in 2018, with a 1.21-fold increase between 2017 and 2020. Interestingly, previous reports indicated that lower extremity amputation decreased between 2000 and 2008, while diabetic patients showed an overall increase in non-traumatic amputations between 2009 and 2015 (20, 23). However, our results revealed a striking increase in the rate of amputation among PAD patients, up to 133-fold in 2020 compared to 2008 in the US.

The demographic profile of PAD is less clear compared to other ASCVD risk factors. Studies have shown that men have almost double the incidence of PAD compared to women based on intermittent claudication (24–26). However, when objective measures [e.g., ankle-brachial index (ABI)] were used, the sex discrepancy was much less drastic (25, 27). Our findings consistently show slight sex differences in PAD prevalence between men and women. However, our analysis showed that 60% of CLI reported between 2008 and 2020 were men. Furthermore, this gap increased across the last decade (the male-to-female ratio = 1.27 in 2008 compared to 1.62 in 2020). This may explain why symptomatic PAD tends to be more common among men. Additionally, the Multi-Ethnic Study of Atherosclerosis (MESA) found that borderline ABI (ABI 0.9–0.99) was more common in women while true PAD (ABI <0.9) tended to be equally represented in both sexes (28–30).

The prevalence of PAD varies heavily based on race and ethnicity. Prior studies showed that Non-Hispanic Blacks tend to have higher rates of PAD, with the highest prevalence after age 50 years in men and 60 years in women (3, 31–34). After the fifth decade, the incidence of PAD among Non-Hispanic Blacks is almost twice as high as that of other races and nearly three times higher in the eighth decade (3). However, a recent study among veterans reported that between 2008 and 2016, the incidence of PAD was less than 10% higher among Non-Hispanic Blacks compared to non-Hispanic whites at the mean age of 60.2 years (19). These inconsistent data are likely due to study design, ABI vs. symptomatic PAD, and different study lengths. Consistently, our study showed that while most PAD cases were reported among non-Hispanic whites, Asians or Pacific Islanders exhibited the most significant rise in PAD between 2008 and 2020. However, CLI was more than 1.5-fold higher among Non-Hispanic Blacks, followed by Native Americans (1.23-fold), compared to Non-Hispanic Whites.

The geographical distribution and trends associated with PAD have been poorly studied. Non-traumatic amputations showed significant regional variations, with higher rates tending to concentrate in the east south-central, west south-central, and south Atlantic regions (southeastern regions) compared to the mountain, New England, and west north-central regions of the US (20). Moreover, amputations were disproportionally higher among Non-Hispanic Blacks in the high amputation regions compared to other races (20). Our study provides the first data regarding the geographical disparity of PAD in the US. As we mentioned earlier, our findings show a significant increase in the incidence of PAD in the western region from 2008 to 2020. Interestingly, we observed that in 2008, the southern region had over twice the incidence of PAD compared to the western region. However, by 2020, the incidence of PAD in the western region had surpassed that of the southern region and the rest of the country.

Although the association between substance use, particularly methamphetamine and cocaine, and coronary artery disease and cerebrovascular diseases has been well studied (14, 17, 35–38), the epidemiological, clinical, and biological aspects of these drugs in relation to PAD are poorly characterized (39, 40). Our study showed that PAD among methamphetamine users is increasing annually, with an almost 6-fold change between 2008 and 2020. The incidence of PAD among methamphetamine users is predominantly concentrated in the western region of the US (60%), which increased significantly between 2008 and 2020. The observed increase in PAD among methamphetamine users in the western region could be a contributing factor to the geographical shift in PAD. In contrast, PAD among cocaine users is more prevalent in the southern region and has shown a modest increase over the last decade. It is noteworthy to state that methamphetamine has a much longer half-life compared to cocaine. Additionally, methamphetamine causes a distinct pattern of endothelial toxicity that affects multiple patterns of endothelial activation compared to cocaine (10, 41).

## Study strengths and limitations

The NIS data have both advantages and disadvantages. Among their strengths, (i) the NIS data on hospitalizations cover an extensive sample size, allowing for robust prevalence and trend analysis; (ii) NIS hospitalization data span numerous years and permit national and state-level estimates; (iii) they provide insightful information about hospital usage and outcomes at the national level in the US; and (iv) the NIS database makes use of standardized diagnosis coding and classification methods, making it possible to analyze trends in hospital usage caused by diseases over time in a consistent and repeatable manner.

Being a cross-sectional study, we could only report temporal association; no definitive conclusion regarding the casualty can be made based on our findings. The selection bias may occur given the retrospective design, ICD-based cohort creation, and inherent errors related to ICD coding. The treatments received by patients, the timing of the interventions, medications, and post-discharge information are unavailable. Moreover, the lack of screening methods to diagnose PAD or CLI among methamphetamine or cocaine users or non-users and the possibility of underestimating prevalence due to insufficient documentation poses further limitations. The analysis in this study focuses solely on PAD and CLI among methamphetamine or cocaine users or non-users and does not discriminate based on other biomarkers or routes of methamphetamine use. Other limitations of the NIS database have been detailed in previous studies (42–44). A larger-scale randomized study followed up on the prospective scale is needed to validate our findings further.

## Conclusion

In conclusion, our paper showed increased PAD in the US with significant geographical and demographic changes over the past decade. We also showed a strong trend of PAD among substance users, especially those who use methamphetamine. There was an increasing trend in PAD-related hospitalizations among methamphetamine and cocaine users for both males and females. Although an overall decreasing trend in CLI-related hospitalization was observed for both genders, an up-trend in CLI was seen among methamphetamine users. These up-trends were more prominent for White, Hispanic & African Americans, and southern and western states, highlighting racial and geographic variations over the study period. Moreover, substance use, especially methamphetamine, needs further investigation to identify risk factors and potential pathologies leading to enhanced PAD.

## Clinical perspective

### Competency in patient care

Certain demographic characteristics such as male sex, methamphetamine use, Pacific Islanders, and Hispanics have higher PAD rates than other populations. Educating at-risk populations and proactive measures might be needed to prevent the rising trends of CLI.

### Translational Outlook

Prospective trials to identify the underlying risk factors in at-risk demographic groups will help guide therapeutic interventions and guidelines.

## Data Availability

The raw data supporting the conclusions of this article will be made available by the authors, without undue reservation.
