# Supplementary material for: Trends in peripheral artery disease and critical limb ischemia hospitalizations among cocaine and methamphetamine users in the United States: a nationwide study
Source: Front Cardiovasc Med. 2024 Jul 3;11:1412867. doi: 10.3389/fcvm.2024.1412867 (PMC11251891; doi:10.3389/fcvm.2024.1412867)
Supplement: Supplementary file 1 [file Table1.docx]

**Supplementary Documents**

**Supplement Table S1. ICD9 and ICD10 codes for PAD, CLI and Amputations.**

| Diagnosis | ICD 10 | ICD 9 |
| --- | --- | --- |
| **Included** |  |  |
| Peripheral artery disease (PAD) | I70 | 440 |
| Critical limb ischemia (CLI) | I70.22- I70.36, I70.42- I70.46, I70.52-I70.55, I70.62- I70.66, I70.72- I70.76 | 440.22-440.24 |
| Amputation | 0X60,0X61,0X62,0X63,0X68,0X69,0X6B,0X6C,0X6D,0X6F,0X6J,0X6K,0X6L,0X6M,0X6N,0X6P,0X6Q,0X6R,0X6S,0X6T,0X6V,0X6W,0Y62,0Y63,0Y64,0Y67,0Y68,0Y6C,0Y6D,0Y6F,0Y6G,0Y6H,0Y6J,0Y6M,0Y6N,0Y6P,0Y6Q,0Y6R,0Y6S,0Y6T,0Y6U,0Y6V,0Y6W,0Y6X,0Y6Y | 840, 841 |

**Supplement Table S2. ICD9 and ICD10 codes.**

| Methamphetamine Dependence | ICD 9 | ICD 10 |
| --- | --- | --- |
| Unspecified | 304.4 | F15.20 |
| Continuous | 304.41 | F15.20 |
| Episodic | 304.42 | F15.20 |
| Remission | 304.43 | F15.21 |
| Methamphetamine Non-dependence | 305.72 | F15.10 |
|  | 305.71 | F15.10 |
|  | 305.72 | F15.10 |
|  | 305.73 | F15.11 |
| Methamphetamine Poisoning | 96972 | T43.621A, T43.622A, T43.623A, T43.624A |
| Cocaine Dependence | ICD 9 | ICD 10 |
| Unspecified | 304.20 | F14.20 |
| Continuous | 304.21 | F14.20 |
| Episodic | 304.22 | F14.20 |
| Remission | 304.23 | F14.21 |
| Nondependent abuse of drugs | 305.6, 305.61, 305.62, 305.63 | F14.10, F14.10, F14.10, F14.11 |
| Poisoning | 97081 | T40.5X1A, T40.5X2A, T40.5X3A, T40.5X4A |
| Obesity | 278x, V77.8 | E66, Z683, Z684, R939, Z6854, 09921 |
| Coronary Artery Disease | 411.x, 412.x, 413.x, 414.x | I25 family |
| Hypertension | 401.x, 402.x, 403.x, 404.x, 405.x | I10, I1150, I1151, I1152, I1158, I1159 |
| Diabetes Mellitus | 250x, 249x | E08-E13 family |
| Heart failure | 250.0x family | I50, I501, I502, I5020, I5021, I5022, I5023, I503, I5030, I5031, I5032, I5033, I504, I5040, I5041, I5042, I5043, I508, I5081, I50810, I50811, I50812, I50813, I50814, I5082, I5083, I5084, I5089, I509 |
| Chronic obstructive pulmonary disease | 491x, 496, 492x, 493x, 494x,495x | J449 |
| Smoker | 305.1, 649.0, V15.82 | F17, Z87.891 |
| Chronic kidney Disease | 585x, | N183, N184, N185, E082, E132, I12, I13 |
| ESRD | 403x, 404x | N186, Z992, Z4931, Z4901 |
| Anemia | 281x, 283.9, 285x, 285x | D50, D51, D52, D53, D55, D56, D57, D58, D59, D60, D61, D62, D63, D64, D46.0, D46.1, D46.2, D46.4, O99.0 |
| Obstructive sleep apnea | 327x | G47.33 |
| History of stroke | V171 | I69.3, Z86.73 |

**Supplement Table S3**. Names of the states in each region (the southern region includes the District of Columbia, the seat of the federal government of the United States).

| Region | States |
| --- | --- |
| Northeast | Connecticut, Maine, Massachusetts, New Hampshire, New Jersey, New York, Pennsylvania, Rhode Island, Vermont |
| Midwest | Illinois, Indiana, Iowa, Kansas, Michigan, Minnesota, Missouri, Nebraska, North Dakota, Ohio, South Dakota, Wisconsin |
| South | Alabama, Arkansas, Delaware, Florida, Georgia, Kentucky, Louisiana, Maryland, Mississippi, North Carolina, Oklahoma, South Carolina, Tennessee, Texas, Virginia, West Virginia  Washington, DC |
| West | Alaska, Arizona, California, Colorado, Hawaii, Idaho, Montana, Nevada, New Mexico, Oregon, Utah, Washington, Wyoming |

**Table S4. Variables used for Multivariate Regression**

| **Variables included in Multivariate Regression Analysis** |
| --- |
| Age |
| Gender |
| Rehab Transfer |
| Resident |
| Elective/Non-elective admissions |
| Hospital Bed Size |
| Hospital Location & Teaching Status |
| Hospital Region |
| Weekend Admission |
| Payer |
| Pulmonary Circulation Disorders |
| Hypertension |
| Chronic Pulmonary Disease |
| OSA |
| Diabetes |
| Hyperlipidemia |
| CKD Stage >3 |
| ESRD |
| Obesity |
| Smoking |
| Prior CVA |
| Hypothyroidism |
| Hemodialysis |
| Family History of CAD |
| Coronary Artery Disease |
| Chronic HF |

Abbreviations: OSA; Obstructive sleep apnea, CKD; Chronic kidney disease, ESRD; End-Stage renal disease, CAD; Coronary artery disease, HF; Heart failure, CVA; Cerebral vascular accident, MI; Myocardial infarction, PCI; Percutaneous coronary intervention, CABG; Coronary artery bypass grafting
